# Effect of Dialysis Modalities on All-Cause Mortality and Cardiovascular Mortality in End-Stage Kidney Disease: A Taiwan Renal Registry Data System (TWRDS) 2005–2012 Study

**DOI:** 10.3390/jpm12101715

**Published:** 2022-10-14

**Authors:** Po-Cheng Su, Cai-Mei Zheng, Chien-Chou Chen, Li-Yun Chiu, Hao-Yun Chang, Meng-Hsu Tsai, Chia-Te Liao, Chih-Chin Kao, Yung-Ho Hsu, Che-Chou Shen, Chih-Cheng Hsu, Mai-Szu Wu, Yen-Chung Lin

**Affiliations:** 1 Department of Internal Medicine, School of Medicine, College of Medicine, Taipei Medical University, Taipei 110, Taiwan; 2 TMU Research Center of Urology and Kidney (TMU-RCUK), Taipei Medical University, Taipei 110, Taiwan; 3 Division of Nephrology, Department of Internal Medicine, Shuang Ho Hospital, Taipei Medical University, New Taipei City 23561, Taiwan; 4 Division of Nephrology, Department of Medicine, Tri-Service General Hospital, National Defense Medical Center, Taipei 114, Taiwan; 5 Department of Medical Education, MacKay Memorial Hospital, No. 92, Sec. 2, Zhongshan N. Rd., Taipei City 10449, Taiwan; 6 Division of General Medicine, Department of Medical Education, Far Eastern Memorial Hospital, New Taipei City 220, Taiwan; 7 Division of Nephrology, Department of Internal Medicine, Taipei Medical University Hospital, 252, Wu-Xing St., Taipei 110, Taiwan; 8 Department of Electrical Engineering, National Taiwan University of Science and Technology, Taipei 10607, Taiwan; 9 Institute of Population Health Sciences, National Health Research Institutes, 35 Keyan Road, Miaoli County 35053, Taiwan

**Keywords:** hemodialysis, peritoneal dialysis, mortality, end-stage kidney disease

## Abstract

Introduction: End-stage kidney disease (ESKD) patients who need renal replacement therapy need to face a dialysis modality decision: the choice between hemodialysis (HD) and peritoneal dialysis (PD). Although the global differences in HD/PD penetration are affected by health-care policies, these two modalities may exert different effects on survival in patients with ESKD. Although Taiwan did not implicate PD as first policy, we still need to compare patients’ outcomes using two modalities in a nation-wise database to determine future patients’ care and health policies. Methods: We used the nationwide Taiwan Renal Registry Data System (TWRDS) database from 2005 to 2012 and included 52,900 patients (48,371 on HD and 4529 on PD) to determine all-cause and cardiovascular mortality among ESKD patients. Results: Age-matched survival probability from all-cause mortality was significantly lower in patients on PD than in those on HD (*p* < 0.05). The adjusted hazard ratios of 3-year and 5-year all-cause and cardiovascular mortality were significantly higher in PD compared with HD. The presence of comorbid conditions including myocardial infarction, coronary artery disease (CAD), diabetes mellitus (DM), hypoalbuminemia, hyperferritinemia and hypophosphatemia was related with significantly higher all-cause and CV mortality in PD patients. No significant difference was noted among younger patients <45 years of age regardless of DM and/or comorbid conditions. Conclusion: Although PD did not have the survival advantage compared to HD in all dialysis populations, PD was related with superior survival in younger non-DM patients, regardless of the presence of comorbidities. Similarly, for younger ESKD patients without the risk of CV disease, both PD and HD would be suitable dialysis modalities.

## 1. Introduction

Taiwan is among the three countries with the highest rates of increase in the number of patients with end-stage kidney disease (ESKD) dependent on long-term dialysis according to the USRDS 2021 report. Not only medical factors and lifestyle concerns but also the dialysis modality itself can affect patient survival [1]. In addition, some countries prefer the “peritoneal dialysis (PD) first” approach, which makes the biggest difference to dialysis penetration globally; the other issues include the percentage of diabetes mellitus (DM), health expenditure as percentage of gross domestic product, and the availability of hemodialysis (HD) facilities [2]. Therefore, whether HD or PD is better remains unclear.

Many studies have compared the survival benefit between PD and HD. HD is associated with an earlier decline of residual renal function [3] and dialysis-induced myocardial and cerebral ischemia [4], whereas PD is associated with the metabolic consequences of excessive absorption of glucose and its degradation products [5], inadequate volume control, and hypokalemia risk [6]. PD can also be technically difficult in patients with impaired physical and cognitive functions [7]. It is associated with a lower risk of mortality than HD in the first few months of dialysis, which seems to diminish with time [8,9,10]. Retrospective studies have revealed that certain survival benefits are influenced by unmeasured confounding factors, such as central venous catheter infection, psychosocial support, nutrition status, or even health-related quality of life [11,12]. Furthermore, it is challenging for physicians to determine the timing of conversion between PD and HD, which mostly depends on dialysis-specific risk factors [13].

Accurate comparisons of survival benefits and appropriate timing of conversion between HD and PD are lacking. Most survival benefits have been demonstrated in observational studies, which tend to have high heterogeneity [14,15]. These studies have provided no strong evidence comparing HD and PD in the latest guidelines [14]. Therefore, we evaluated and compared the adjusted survival benefit of HD and PD by using a nationwide cohort and multivariate Cox regression models.

## 2. Methods

The Taipei Medical University Institutional Review Board approved this study (No. N202201106) and waived the requirement for obtaining informed consent, and all applicable regulations were met. This study was conducted in accordance with the Declaration of Helsinki 1975, as revised in 2013.

### 2.1. Taiwan Renal Registry Data System

The Taiwan Renal Registry Data System (TWRDS) was established in 1987, and all dialysis units in Taiwan provide their patients’ clinical information and all laboratory reports to TWRDS on a quarterly basis. From 1997 onwards, complete information on patients’ comorbidities, such as the history of myocardial infarction (MI), coronary artery disease (CAD), patients’ rehabilitation status, etc. as well as dialysis adequacy indices, biochemical and hematological parameters, hepatitis serological results, information on medication prescriptions, anemia and mineral bone indices were recruited. Based on TWRDS data, the Taiwan Society of Nephrology develops annual guidelines to improve individual patients’ health outcomes and to issue reimbursement from the government. This government reimbursement provides only a very small portion of incentives (5%) for dialysis centers and is separated from medical reimbursement provided by Taiwan’s National Health Insurance (NHI) for overall health expenditures. Hence, the TWRDS data represent continual dialysis quality control at the national level.

### 2.2. Patient Enrollment

**Figure 1:**
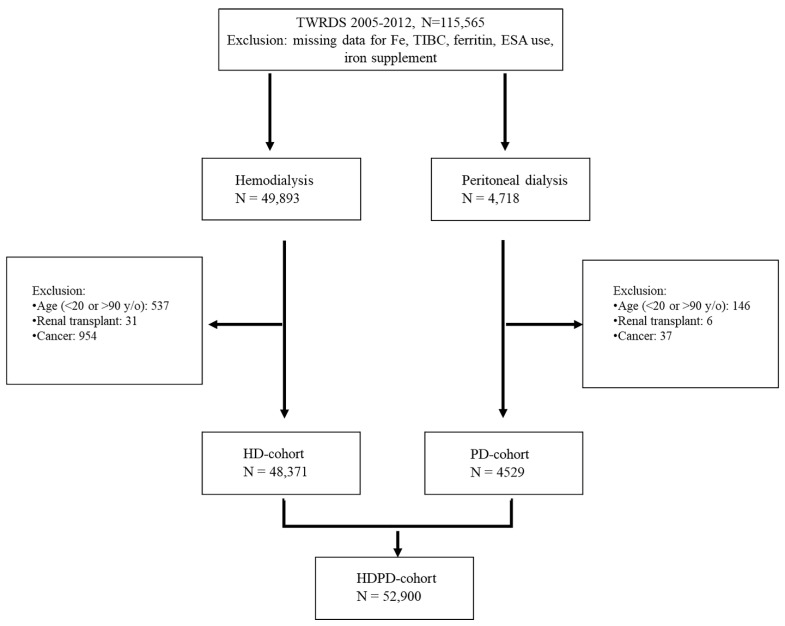
Flow chart of the whole population (N = 52,900).

The data of 115,565 patients from 2005 to 2012 were extracted from the TWRDS. After excluding patients with missing data for Fe^2+^, TIBC, ferritin, ESA use, and iron supplements; age <20 or >90 years; a history of renal transplantation; and cancer, 52,900 patients (48,371 on HD and 4529 on PD) were included (Figure 1).

### 2.3. Statistical Analysis

Continuous variables are expressed as means (standard deviations (SDs)), medians (ranges), or frequencies (percentages), and categorical variables are expressed as proportions. One-way ANOVA was used to compare continuous variables because a normal distribution was assumed in this big data analysis, and the chi-square test was used to compare nominal variables. We used the log-rank test for Kaplan–Meier analysis. A two-tailed *p* < 0.05 was set as the level of significance. We performed Cox regression analysis to estimate the hazard ratios (HR) of 3-year or 5-year all-cause or cardiovascular (CV) mortality. The case-mix adjusted model included the following confounding factors: age, sex, DM, hypertension, myocardial infarction (MI), coronary artery disease (CAD), and biochemistries including albumin, hemoglobin, calcium, phosphate, iron saturation, ferritin, and alkaline phosphatase. Crude and adjusted risks of mortality were estimated in subgroup analysis according to age, DM, and comorbidities. All statistical analyses were performed using SPSS version 17.0 (SPSS, Chicago, IL, USA) and SAS version 9.1 (SAS Institute, Cary, NC, USA).

## 3. Results

### 3.1. Baseline Characteristics

Table 1 presents the baseline characteristics of patients on PD and HD, including age, blood count, and electrolyte and hormone levels. Multiple logistic regression analysis revealed that patients on PD were significantly younger, less anemic, and less hyperglycemic than those on HD. Furthermore, patients on HD were more likely to have higher albumin levels and reduced P and iPTH levels than patients on PD. WBC counts and alkaline-P, triglyceride, uric acid, and Ca levels were not significantly different between the groups.

### 3.2. Kaplan–Meier Curves

**Figure 2:**
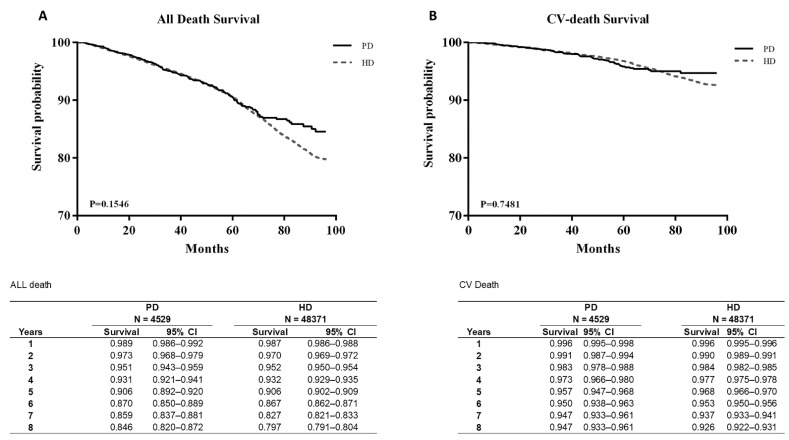
Survival curves between all-death survival (**A**) and CV-death survival (**B**).

Figure 2A presents the survival curves of all-cause mortality and CV mortality, and Figure 2B plots the two curves after age matching. Both figures cover the same 8-year period of 2005–2012. The survival probability of all-cause mortality between PD and HD was similar up to 70 months, and the survival probability was non-significantly higher in patients on PD than in those on HD after 70 months (Figure 2A).

**Figure 3:**
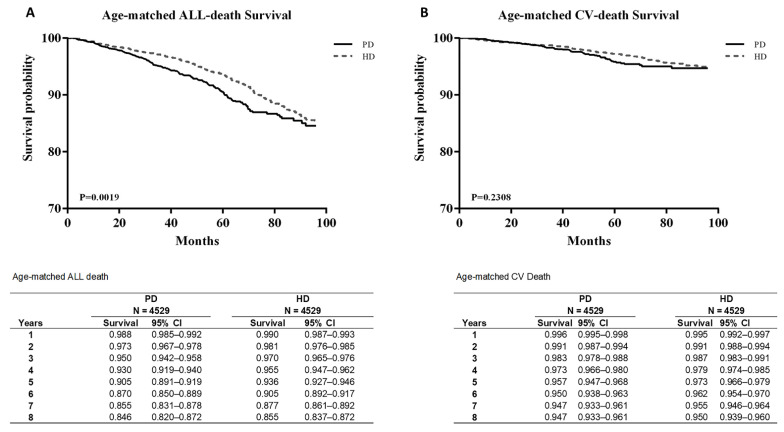
Age-matched survival curves between all-death survival (**A**) and CV-death survival (**B**).

CV mortality was also not significantly different between the groups. After age matching, the survival probability from all-cause mortality was significantly lower in patients on PD than in those on HD (*p* < 0.05). However, CV mortality showed no significant difference even after age matching (Figure 3B).

### 3.3. Hazard Ratio of Mortality between HD and PD in the Multivariate Cox Regression Model

We further analyzed the factors that influence mortality after correcting the hazard ratio in three multivariate Cox regression models. In model 1, we considered basic profiles such as age, male, and PD versus HD. Model 2 included comorbidities such as hypertension, MI, CAD, and DM. Finally, model 3 included laboratory data such as transferrin saturation and ferritin levels.

Table 2A presents the hazard ratios of 3-year all-cause mortality in the overall cohort. PD versus HD, age, and male sex were all significantly different in all three models. Hb < 10 g/dL versus Hb 10–12 g/dL, hypertension, MI, CAD, and DM in model 2 and hypertension, DM, albumin < 3.5 mg/dL, albumin > 4.0 mg/dL, and ferritin < 100 mg/dL in model 3 were also significantly different.

Table 3 present the hazard ratios of 5-year all-cause and CV mortality, respectively, in the overall cohort. Compared with the results in Table 2, they were different in model 2. Table 3 only shows PD versus HD; age, male sex, CAD, and DM showed significant differences in model 2. Compared with the results in Table 2, they were different in model 3. As presented in Table 3, albumin >4.0 mg/dL exhibited a significant difference in model 3.

### 3.4. DM and Comorbidities: Subgroup Analysis

As shown in Table 4, patients on HD and PD were subdivided into four groups: with or without comorbidities and with or without DM. Regardless of the presence of comorbidities, including DM, the death rate in the HD and PD groups increased with patient age. The crude relative risk (RR) was higher in patients on PD than in patients on HD in all of the following subgroups: age ≥65 years without DM/comorbidities (crude RR, 1.42; 95% confidence interval (CI), 1.06–1.90), age of 45–64 years with DM but without comorbidities (crude RR, 1.57; 95% CI, 1.15–2.15), age of ≥65 years with DM but without comorbidities (crude RR, 1.47; 95% CI, 1.02–1.95), age of 45–64 years without DM but with comorbidities (crude RR, 1.75; 95% CI, 1.03–2.99), and age ≥65 with both DM and comorbidities (crude RR, 2.26; 95% CI, 1.43–3.52). However, the adjusted RR was significantly higher in patients on PD than in patients on HD in only two of those subgroups: age of 45–64 years with DM but without comorbidities (adjusted RR, 1.44; 95% CI, 1.05–1.97) and age ≥65 years with both DM and comorbidities (adjusted RR, 1.96; 95% CI, 1.24–3.11).

## 4. Discussion

In a previous study, DM, age, and baseline comorbidities were the key factors that influenced the RR of death between PD and HD [16]. In Taiwan, HD is preferred to PD among patients with ESKD, which is similar to the trend in other countries [16,17,18]. However, we hypothesized that survival outcomes may not be identical between the two groups, and advancements such as more biocompatible solutions might make PD a more favorable choice [14]. In this study, we conducted a detailed comparison between PD and HD outcomes. To the best of our knowledge, it has been a long time since the last large-scale native study has been conducted in Taiwan. We therefore used TWRDS in our study design for comparing PD and HD. Our study comprehensively identified the factors that influence the RR of death between HD and PD, including the cause of ESKD (DM vs. non-DM), age (18–44, 45–64, and ≥65 years), baseline comorbidity (none vs. one or more comorbidities), and laboratory data. We also performed in-depth analyses of CV and all-cause mortality over 3-year and 5-year time horizons, which might solve the potential limitations of previous studies.

Although the survival probability in patients on PD was non-significantly higher than that in patients on HD after 70 months; age-matched analysis revealed that the survival probability in patients on PD was significantly lower than that in patients on HD. Comparing all-cause mortality in Table 2A and Table 3A, significant HRs were obtained for hypertension, MI, CAD, and DM among all models in the 5-year analysis, but significant HRs were only found for hypertension and DM among all models in the 3-year analysis. Next, we compared CV mortality, and only DM contributed to the significant differences. Our study indicated that PD, age, and male sex were always risk factors for mortality. PD increased the risk of all-cause mortality, which indicated that patients with more comorbidities such as hypertension, MI, CAD, and DM might have higher risks of using PD, and the risk was higher if the patients had longer duration of PD use. However, without the risk of CV disease, both PD and HD will be suitable modalities.

Our results are consistent with those of previous studies. Weinhandl et al. [19] reported that PD was associated with better survival than HD among patients without CV disease, whereas HD was associated with improved survival among patients with CV disease and diabetes. Liem et al. [20] concluded that the survival advantage for PD compared with HD decreased over time, with age, and in the presence of diabetes as a primary disease. Kim et al. [21] also concluded that in older patients (aged ≥55 years), PD was consistently associated with a higher mortality rate with the consideration of comorbidities. McDonald et al. [21,22] revealed that among younger patients (aged <60 years) without comorbidities, the PD group exhibited better survival over 90–365 days, and that this initial survival advantage persisted for 4 years. Collins et al. [23] concluded that within the first 2 years of therapy, short-term CAPD/CCPD was associated with better outcomes than HD. Although several studies have been analyzed different survival periods [24,25], their conclusions are similar: patients under PD who are younger or had no or few comorbidities may have better outcomes over the short term.

In our subgroup analyses of the influence of DM and comorbidities on dialysis modality, patients aged 20–44 years with DM but without comorbidities and those aged 20–44 with both DM and comorbidities had adjusted RRs (PD vs. HD) of 0.95 (95% CI, 0.37–2.47) and 0.53 (95% CI, 0.07–4.03), respectively (Table 4). However, older patients in the other groups exhibited higher RRs. Our results correspond to those of Vonesh et al. [11]. They concluded that PD was associated with an increased mortality risk in patients with DM aged ≥45 years. However, HD was associated with an increased mortality risk in the population comprising non-DM patients and younger patients with DM but without comorbidities. Our data did not indicate a significant difference between PD and HD, which was likely due to the low amounts of patients’ data included in these two subgroups, with patient percentages of only 1% and 2% for each dialysis type. Further large-scale analyses should focus on younger patients with DM undergoing PD or HD. Other studies have also supported our findings. In a Korean study, Kim et al. [21] reported that regardless of covariates, the survival rate was comparable between younger patients on PD and HD. van de Luijtgaarden et al. [26] also reported that overall, patients starting on PD, especially those without comorbidities, had survival benefits. Collins et al. [23] reported a lower mortality risk in younger patients (<55 years old) on CAPD/CCPD without diabetes (men: RR, 0.61; 95% CI, 0.59–0.66 and women: RR, 0.72; 95% CI, 0.67–0.77) or with diabetes (men: RR, 0.88; 95% CI 0.82–0.94 and women: RR, 0.86; 95% CI 0.81–0.92).

This study has some limitations. It was a retrospective observational study, and its generalizability is still required. The most important factor, actual clearance levels is not justifiable in such retrospective comparison between two modalities. Further, no adjustments were made during selection of the type of dialysis, such as duration of CKD, timing of nephrologists’ care, and patients’ family support for their daily HD or PD. Individualized data important for patient and family care, including various socioeconomic factors, were unavailable and thus could not be adjusted in this study. We believe that future prospective randomized studies that include younger patients can address these limitations.

In conclusion, we proved that PD did not revealed a survival benefit as compared with HD, especially in the elder population. Both PD and HD might be suitable for younger patients <45 years old with or without DM and regardless of comorbid conditions and underlying CV risk factors. Thus, individual evaluation and explanation is important for dialysis modality choice in each and every ESKD patient.

## Figures and Tables

**Table 1:** Characteristics of the population (N = 52,900).

	PD (N = 4529)	HD (N = 48,371)	
Variables	Means ± SD	Means ± SD	*p*-Value
Age (yr)	53.74 ± 14.68	62.02 ± 13.54	<0.001
W.B.C. (×1000/μL)	7.07 ± 2.77	7.11 ± 3.53	0.066
R.B.C. (×10^6^/μL)	3.40 ± 0.58	3.42 ± 3.69	<0.001
Hbc (g/dL)	10.08 ± 1.46	9.80 ± 1.40	<0.001
MCV (fL)	89.25 ± 6.96	91.40 ± 7.22	<0.001
Platelet (×1000/μL)	220.37± 79.85	202.08 ± 78.56	<0.001
Albumin (gm/dL)	3.65 ± 0.50	3.72 ± 0.47	<0.001
A.S.T.[GOT] (IU/L)	23.58 ± 16.64	22.51 ± 15.92	<0.001
Alkaline-P (IU/L)	121.44 ± 107.90	121.93 ± 102.59	0.626
Total Bilirubin (mg/dL)	0.39 ± 0.52	0.45 ± 0.65	<0.001
Cholesterol (mg/dL)	194.63 ± 49.19	172.09 ± 0.65	<0.001
Triglyceride(mg/dL)	153.08 ± 112.25	154.40 ± 114.58	0.572
Glucose[AC] (mg/dL)	127.34 ± 69.24	152.24 ± 83.17	<0.001
BUN (mg/dL)	68.79 ± 23.57	72.19 ± 22.26	<0.001
Creatinine (mg/dL)	9.51 ± 3.02	9.01 ± 2.71	<0.001
Uric acid (mg/dL)	7.20 ± 1.53	7.22 ± 1.51	0.117
Na (meq/L)	137.53 ± 4.20	137.22 ± 3.89	<0.001
K (meq/L)	4.01 ± 0.73	4.55 ± 0.84	<0.001
Ca (mg/dL)	9.15 ± 0.92	9.15 ± 0.92	0.0307
P (mg/dL)	5.00 ± 1.47	4.84 ± 1.61	<0.001
CCr (mL/min)	5.92 ± 2.21	6.67 ± 2.48	<0.001
Ferritin (ng/mL)	316.60 ± 240.21	365.65 ± 246.22	<0.001
Tranferrin saturation (%)	27.90 ± 14.29	26.93 ± 13.21	<0.001
intact-PTH (pg/mL)	307.27 ± 230.19	230.36 ± 204.18	<0.001

**Table 2:** (**A**) Hazard ratio (HR) of 3-year all-cause mortality in the whole population. (**B**) Hazard ratio (HR) of 3-year cardiovascular mortality in the whole population (N = 52,900).

(A)	Model 1			Model 2			Model 3		
	HR	95% CI	*p*-Value	HR	95% CI	*p*-Value	HR	95% CI	*p*-Value
PD vs. HD	1.58	1.34–1.88	<.0001	1.46	1.23–1.74	<0.001	1.49	1.25–1.77	<0.001
Age	1.06	1.06–1.07	<.0001	1.06	1.05–1.06	<0.001	1.06	1.05–1.06	<0.001
Gender (male)	1.18	1.06–1.27	<0.001	1.15	1.05–1.26	<0.001	1.18	1.07–1.29	<0.001
Hb < 10 g/dL				1.21	1.10–1.33	<0.001	0.98	0.89–1.08	0.66
Hb between 10–12 g/dL				1			1		
Hypertension				0.83	0.74–0.93	<0.01	0.85	0.75–0.95	<0.01
Myocardial infarction				1.64	1.21–2.22	<0.01	1.40	0.69–2.83	0.35
Coronary artery disease				1.74	1.37–2.21	<0.001	1.49	0.85–2.61	0.17
Diabetes mellitus				1.83	1.67–2.01	<0.001	1.79	1.46–2.19	<0.001
Albumin									
Alb < 3.5 mg/dL							1.64	1.48–1.82	<0.001
Alb = 3.5–4.0 mg/dL							1		
Alb > 4.0 mg/dL							0.63	0.54–0.72	<0.001
Fe Saturation (%)							0.99	0.98–1.00	0.22
Ferritin level									
<100 mg/dL							0.81	0.70–0.94	<0.001
100–499 mg/dL							1	-	-
400–800 mg/dL							1.06	0.94–1.19	0.36
>800 mg/dL							1.22	1.02–1.45	0.03
**(B)**	**Model 1**			**Model 2**			**Model 3**		
	**HR**	**95% CI**	***p*-Value**	**HR**	**95% CI**	***p*-Value**	**HR**	**95% CI**	***p*-Value**
PD vs. HD	1.51	1.12–2.04	<0.01	1.46	1.08–1.98	0.01	1.61	1.15–2.24	<0.01
Age	1.06	1.05–1.07	<0.001	1.06	1.05–1.07	<0.001	1.06	1.05–1.07	<0.001
Gender (male)	1.18	1.01–1.38	0.04	1.18	1.00–1.38	0.04	1.18	0.97–1.45	0.10
Hb < 10 g/dL				1.04	0.96–1.12	0.36	1.05	0.80–1.35	0.66
Hb 10–12 g/dL				1			1		
Hypertension				0.94	0.77–1.14	0.50	0.88	0.69–1.23	0.29
Myocardial infarction				1.24	0.70–2.20	0.13	1.40	0.69–2.83	0.35
Coronary artery disease				1.51	0.90–2.19	0.13	1.49	0.85–2.61	0.35
Diabetes mellitus				1.86	1.58–2.19	<0.001	1.79	1.46–2.20	<0.001
Alb < 3.5 mg/dL							1.49	1.19–1.88	<0.001
Alb = 3.5–4.0 mg/dL							1	-	-
Alb > 4.0 mg/dL							0.82	0.62–1.10	0.16
Ca < 8.5 (mg/dL)							0.97	0.75–1.26	0.84
Ca 8.5–10.2 (mg/dL)							1	-	-
Ca > 10.2 (mg/dL)							0.75	0.52–1.08	0.12
P < 2.5 (mg/dL)							1.35	0.83–1.38	0.12
P 2.5–4.5 (mg/dL)							1	-	-
P > 4.5 (mg/dL)							1.02	0.82–1.26	0.86
ALK-P							1.001	1–1.002	<0.01
Intact PTH < 150 pg/dL							1.07	0.84–1.36	0.57
Intact PTH 150–300 pg/dL							1	-	-
Intact PTH 300–450 pg/dL							0.84	0.591.20	0.34
Intact PTH > 450 pg/dL							0.93	0.65–1.33	0.70

PD: peritoneal dialysis; HD: hemodialysis; Alb: serum albumin; Ca: serum calcium; ALK-P: alkaline phosphatase; PTH: parathyroid hormone.

**Table 3:** (**A**) Hazard ratio (HR) of 5-year all-cause mortality in the whole population (N = 52,900). (**B**) Hazard ratio (HR) of 5-year cardiovascular mortality in the whole population (N = 52,900).

(A)	Model 1			Model 2			Model 3		
	HR	95% CI	*p*-Value	HR	95% CI	*p*-Value	HR	95% CI	*p*-Value
PD vs. HD	1.59	1.38–1.84	<0.001	1.48	1.28–1.71	<0.001	1.49	1.25–1.77	<0.001
Age	1.07	1.06–1.07	<0.001	1.06	1.06–1.06	<0.001	1.06	1.05–1.06	<0.001
Gender (male)	1.19	1.10–1.28	<0.001	1.18	1.09–1.27	<0.001	1.18	1.07–1.29	<0.001
AnemiaHb < 10 g/dL				1.04	0.96–1.12	0.36	0.98	0.89–1.08	0.66
Hb between 10–12 g/dL				1			1		
Hypertension				0.87	0.79–0.96	0.004	0.85	0.75–0.95	<0.01
Myocardial infarction				1.51	1.18–1.94	0.001	1.40	0.69–2.83	0.35
Coronary artery disease				1.62	1.32–1.99	<0.0001	1.49	0.85–2.61	0.17
Diabetes mellitus				1.80	1.66–1.94	<0.0001	1.79	1.46–2.19	<0.001
Albumin									
Alb < 3.5 mg/dL							1.64	1.51–1.79	<0.001
Alb = 3.5–4.0 mg/dL							1	-	-
Alb > 4.0 mg/dL							0.63	0.56–0.70	<0.001
Fe Saturation (%)							0.99	0.98–0.99	0.04
Ferritin									
<100 mg/dL							0.82	0.73–0.92	<0.001
100–499 mg/dL							1	-	-
400–800 mg/dL							1.00	0.91–1.11	0.95
>800 mg/dL							1.06	0.92–1.24	0.42
**(B)**	**Model 1**			**Model 2**			**Model 3**		
	**HR**	**95% CI**	***p*-Value**	**HR**	**95% CI**	***p*-Value**	**HR**	**95% CI**	***p*-Value**
PD vs. HD	1.59	1.38–1.84	<0.001	1.48	1.28–1.71	<0.001	1.70	1.29–2.24	<0.001
Age	1.06	1.06–1.07	<0.001	1.06	1.06–1.06	<0.001	1.06	1.05–1.07	<0.001
Gender (male)	1.22	1.08–1.39	<0.01	1.18	1.09–1.27	<0.001	1.2	1.02–1.41	0.03
Hb < 10 g/dL vs. Hb 10–12 g/dL)				1.04	0.96–1.12	0.36	1.07	0.90–1.28	0.42
Hypertension				0.87	0.79–0.96	<0.01	0.88	0.72–1.07	0.21
Myocardial infarction				1.51	1.18–1.94	<0.01	1.23	0.66–2.31	0.51
Coronary artery disease				1.62	1.32–1.91	<0.001	1.50	0.93–2.41	0.10
Diabetes mellitus				1.80	1.66–1.94	<0.001	2.03	1.71–2.40	<0.001
Alb < 3.5 mg/dL							1.49	1.23–1.79	<0.001
Alb = 3.5–4.0 mg/dL							1	-	-
Alb > 4.0 mg/dL							0.88	0.70–1.10	0.25
Ca < 8.5 (mg/dL)							0.91	0.73–1.13	0.37
Ca 8.5–10.2 (mg/dL)							1	-	-
Ca > 10.2 (mg/dL)							0.92	0.70–1.21	0.56
P < 2.5 (mg/dL)							1.18	0.84–1.64	0.34
P 2.5–4.5 (mg/dL)							1	-	-
P > 4.5 (mg/dL)							0.96	0.81–1.14	0.67
ALK-P							1.001	1–1.002	<0.01
Intact PTH < 150 pg/dL							1	0.82–1.22	1.00
Intact PTH 150–300 pg/dL							1	-	-
Intact PTH 300–450 pg/dL							0.89	0.67–1.18	0.42
Intact PTH > 450 pg/dL							0.98	0.74–1.31	0.912

PD: peritoneal dialysis; HD: hemodialysis; Alb: serum albumin; Ca: serum calcium; ALK-P: alkaline phosphatase; PTH: parathyroid hormone.

**Table 4:** Mortality risk by stratifying diabetes mellitus, comorbid condition, and comorbidities.

Comorbid Condition	DM	Age	HD			PD				Relative Risk (PD vs. HD)	
			Deaths	Death Rate (%)	95% CI	Deaths	Death Rate (%)	95% CI	Patient %	Crud RR	Adjust RR
None	Non_DM	20–44	59	0.42	0.32–0.54	13	0.47	0.27–0.81	7%	1.41 (0.75–2.58)	1.35 (0.72–2.50)
		45–64	342	0.96	0.86–1.07	34	0.92	0.66–1.29	17%	1.16 (0.82–1.67)	1.12 (0.78–1.60)
		≥65	904	3.11	2.91–3.32	47	3.82	2.87–5.09	18%	1.42 (1.06–1.90) *	1.28 (0.95–1.71)
	DM	20–44	47	1.61	1.21–2.15	5	1.45	0.6–3.49	2%	1.22 (0.48–3.12)	0.95 (0.37–2.47)
		45–64	506	2.21	2.03–2.41	44	2.84	2.11–3.82	14%	1.57 (1.15–2.15) *	1.44 (1.05–1.97) *
		≥65	796	4.36	4.06–4.67	38	5.29	3.85–7.28	13%	1.47 (1.02–1.95) *	1.30 (0.93–1.81)
One or more	Non_DM	20–44	19	0.37	0.24–0.58	3	0.40	0.13–1.24	2%	1.41 (0.41–4.88)	1.49 (0.40–5.55)
		45–64	170	1.29	1.11–1.5	15	1.57	0.95–2.61	6%	1.75 (1.03–2.99) *	1.68 (0.98–2.86)
		≥65	336	3.44	3.09–3.82	8	3.31	1.66–6.63	6%	1.33 (0.66–2.70)	1.11 (0.55–2.27)
	DM	20–44	21	1.53	1–2.35	1	0.63	0.09–4.46	1%	0.58 (0.07–4.36)	0.53 (0.07–4.03)
		45–64	255	2.39	2.12–2.71	10	1.93	1.04–3.59	7%	1.15 (0.61–2.18)	1.02 (0.54–1.94)
		≥65	326	4.06	3.64~4.53	20	7.60	4.9~11.78	6%	2.26 (1.43–3.52) **	1.96 (1.24–3.11) **

* *p* < 0.05, ** *p* < 0.001, Adjust for gender, albumin, hemoglobin.

## Data Availability

No new data were created or analyzed in this study. Data sharing is not applicable to this article.
